# Intestinal mucosal microbiota mediate amino acid metabolism involved in the gastrointestinal adaptability to cold and humid environmental stress in mice

**DOI:** 10.1186/s12934-024-02307-2

**Published:** 2024-01-24

**Authors:** Chen-Yang Zhang, Xin-Xin Peng, Yi Wu, Mai-Jiao Peng, Tiao-Hao Liu, Zhou-Jin Tan

**Affiliations:** 1 College of Traditional Chinese Medicine, Hunan University of Chinese Medicine, Changsha, China; 2 Department of Pediatrics, The First Affiliated Hospital of Hunan University of Chinese Medicine, Changsha, China; 3 College of Pharmacy, Hunan University of Chinese Medicine, Changsha, China; 4 Department of Gastroenterology, Affiliated Hospital of Jiangnan University, Wuxi, China

**Keywords:** Cold and humid environmental stress, Gastrointestinal (GI) disorders, Intestinal mucus barrier, Intestinal mucosal microbiota, Multi-omics, Amino acid

## Abstract

Growing evidence has demonstrated that cold and humid environmental stress triggers gastrointestinal (GI) disorders. In this study, we explored the effects of intestinal microbiota homeostasis on the intestinal mucus barrier and GI disorders by cold and humid environmental stress. Moreover, the inner link between the intestinal mucosal microbiota and metabolites in mice with cold and humid environmental stress was interpreted by integrative analysis of PacBio HiFi sequencing microbial genomics and targeted metabolomics. In the current study, we found (1) after the cold and wet cold and humid environmental stress intervened in the intestinal microbiota disorder and homeostasis mice respectively, the bacterial culturing and fluorescein diacetate (FDA) microbial activity detection of intestinal microbiota including feces, intestinal contents, and intestinal mucosa suggested that the cold and humid environmental stress decreased the colony of culturable bacteria and microbial activity, in which intestinal microbiota disorder aggravated the injury of the intestinal mucus barrier and the GI symptoms related to cold and humid environmental stress; (2) the serum amino acid transferases such as glutamate pyruvic transa (GPT), and glutamic oxaloacetic transaminase (GOT) in cold and humid environmental stressed mice increased significantly, indicating that the intestinal microbiota adapted to cold and humid environmental stress by regulating the host's amino acid metabolism; (3) the integrative analysis of multi-omics illustrated a prediction model based on the microbiota *Lactobacillus reuteri* abundance and host amino acid level that can predict intestinal mucoprotein Muc2 with an adjusted R2 of 75.0%. In conclusion, the cold and humid environmental stress regulates the neurotransmitter amino acids metabolic function both in intestinal mucosal microbiota and host serum by adjusting the composition of the dominant bacterial population *Lactobacillus reuteri*, which contributes to the intestinal mucus barrier injury and GI disorders caused by cold and humid environmental stress.

**Supplementary Information:**

The online version contains supplementary material available at 10.1186/s12934-024-02307-2.

## Introduction

The latest research shows that clinical hypothermia therapy can activate the body's heat production system to obtain therapeutic benefits [1]. However, hypothermia brings a crisis to the immune resistance in patients in intensive care or postoperative. Stress caused by cold and humid stimuli leads to the emergence of strong defense systems, which highly simulate the pathophysiology prevalent in patients in intensive care or postoperative [2]. When cold and humid stressors are repeated, the organism adaptive responses are dysregulated and result in disease, causing GI disorders [3]. Intestinal microbiota may be involved in the host’s adaptability to environmental stress, for temperature is a core factor that controls microbial growth [4]. But the impact of cold and humid stressors on the microbiome and GI disorders remains to be fully explored. In this study, the microecological mechanism of cold and humid stress was explored to look for strategies for the side effects of GI disorders caused by clinical hypothermia therapy.

Cold and humid exposure increased intestinal permeability and resulted in intestinal barrier dysfunction contributing to decreased intestinal microbiota diversity [5]. The mucosal layer on the intestinal wall forms an intestinal barrier. The mucus layer, a giant gel network structure mainly formed by the dehydration of secreted mucoprotein 2 (Muc2), is attached to the intestinal mucosa, to prevent the entry of toxic substances and pathogenic microorganisms into the intestinal cavity, which is the first prerequisite for maintaining the physiological function of the intestine [6]. The mucin glycoprotein family, characterized by glycosylation, is mostly composed of oligosaccharides or carbohydrates formed by glycan chains, so it can be degraded by microbial enzymes that can utilize glycans and glycoproteins [7]. The mucin microbial degradation is an important process for both normal microbial gut colonization and diseases such as inflammatory bowel disease [8]. Moreover, the intestinal mucus barrier is the first space for the interaction between intestinal microbiota and the host, suggesting the attention demand to the contribution of intestinal mucosal microbiota to cold and humid environmental stress.

Temperature, whether external or internal, is the core factor controlling the growth of microorganisms, it drives the change of microbial composition and directly regulates the growth and virulence of pathogenic microorganisms in the GI tract [4]. Cold stress can reduce the activity of bacterial enzymes, but it is worth noting that the microbiota not only responds to low temperature but also its metabolites can activate brown adipose tissue (BAT) and affect the thermogenesis of the host body [9], meaning the intervention factors of external environmental stress on the microbiota in vivo are complicated and uncharted. Cold exposure can activate the metabolic process required to promote thermogenesis, and the cold-acclimatized metabolic process involves amino acids metabolism [10]. A study found that alanine/serine/cysteine transporter-1 (ASC-1) relies on the consumption of serine, cysteine, and glycine to effectively stimulate the heat production of human adipocytes [11]. However, there are few studies on the correlation between amino acids involved in intestinal microbiota and intestinal mucus barrier injury. Our previous studies have found that tryptophan metabolism of intestinal microbiota, especially in the intestinal mucosa, is involved in the formation of GI disorders [12, 13]. Considering that the injury of the intestinal mucus barrier is an early manifestation of GI disorders [14], we hypothesized that intestinal mucosal microbiota interacts with amino acid metabolism to mediate the intestinal mucus barrier in response to cold and humid environmental stress. Here, according to our previous studies [15–17], we prepared intestinal microbiota disorder mice model to confirm the role of intestinal microbiota by analyzing whether intestinal microbiota disorder will aggravate intestinal mucosal damage and GI disorders caused by cold and humid environmental stress. Moreover, the current study discussed the potential link between the intestinal mucosal microbiota and metabolites in mice with cold and humid environmental stress was interpreted by integrative analysis of PacBio HiFi sequencing microbial genomics and targeted metabolomics.

## Materials and methods

### Animals

Four-week-old SPF Kunming mice (n = 28; average weight, 20 g) were gained from the Slack Jingda Experimental Animal Co, Ltd. (Hunan, China). According to our previous research, male mice are used in this experiment [18]. The mice were maintained in a shielded environment at the Animal Experiment Center of the Hunan University of Chinese Medicine (Hunan, China, SYXK [Xiang] 2015-0003). Each mouse was housed individually under standard controlled laboratory conditions (12/12 dark–light cycle, temperature of 21 ± 2 °C, humidity of 45 ± 10%), with ad libitum access to food and water.

### Experiment design

**Fig. 1:**
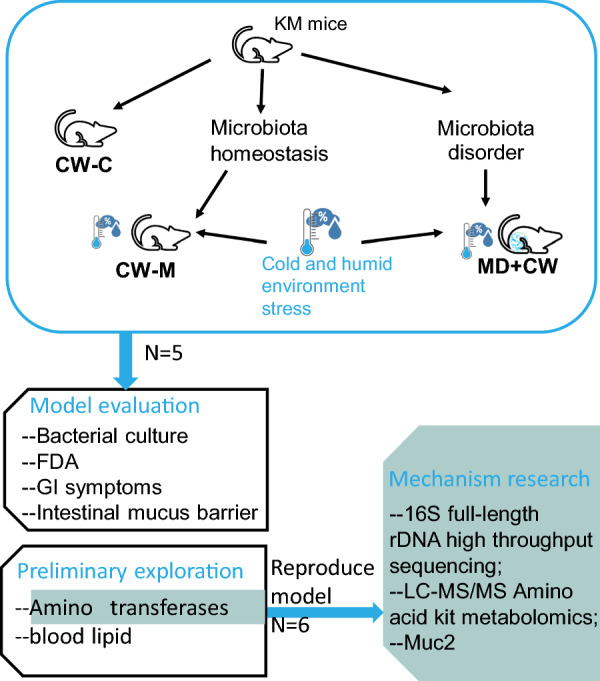
Animal grouping and treatments

The animal experiment was designed to intervene in intestinal microbiota homeostasis in the host and determine the role of intestinal microbiota homeostasis in host adaptive regulation of GI disorders caused by cold and humid environmental stress. After a week to habituate freely for acclimatization, all animal randomized grouping and treatments are described in Fig. 1. Firstly, to confirm the role of intestinal microbiota by the intestinal microbiota disorder, according to our previous studies [15–17] the mice were treated with antibiotics mixture to cause the intestinal microbiota disorder. The other mice were given the same dose of normal saline by gavage. The cold and humid environmental stress intervention in mice with intestinal microbiota homeostasis and intestinal microbiota disorder, forming the cold and humid environmental stress treatment group (CW-M) and microbiota disorder + cold and humid environmental stress treatment group (MD + CW), respectively. The cold and humid environmental stress intervention method: the mice were maintained in an artificial climate room from a.m. 8:00 to 12:00 with a temperature of 4 ± 0.5 °C, and a humidity of 90 ± 2%, and in the remaining time, the model mice were fed in the normal shielded environment, for continual 7 days. In the meantime, the normal control group (CW-C) mice were fed in the normal shielded environment. Then, to study the metabolic mechanism of intestinal mucosal microbiota by reproducing cold and humid environmental stress animal models.

During the artificial climate room treatment, the feces of the 3 groups of mice were collected at the same time every day to determine moisture content and calculate the diarrhea index [19, 20].

1 $${\text{Diarrhea index }} = {\text{ l1 }}*{\text{ L}}$$

where l1 is loose stool rate (number of loose feces per mouse/total feces) and L is loose stool grade. The area of dirt formed by dilute fecal pollution filter paper is classified into 4 loose stool grades. Grade 1: pollution diameter < 1 cm; Grade 2: 1 cm ≤ pollution diameter < 2 cm; Level 3: 2 cm ≤ pollution diameter ≤ 3 cm; Grade 4: pollution diameter > 3 cm.

Body mass and food intakes of all experimental mice were measured every day at baseline until the end of the study, to calculate the food efficiency ratio[21, 22].

2 $${\text{Food efficiency ratio }} = {\text{ M1 }}/{\text{ M }}*{ 1}00$$

where M1 is average daily weight gain (g) and M is average daily food intake(g).

After the treatment, mice were fasted for 12 h before taking samples, and the blood and intestinal tissue samples were extracted.

### Surgical sampling of the intestinal mucosa

#### Bacterial culturing

Fecal samples of mice were collected at an interval of 1 day until the end of the study, and samples of intestinal contents from the duodenum to the ileocecal segment were extracted, which were used for bacterial culture. Fully mix the same kind of samples in the same group, weigh 0.5 g of mixed sample, dilute with 30 mL sterile distilled water, and then shake at 120 R/min for 30 min to prepare a bacterial solution. Beef extract peptone AGAR medium was used for the bacterial culture. The bacterial solution was diluted with appropriate dilution and coated with the plate. Finally, the Petri dishes were cultured in the 37 ℃ incubator and anaerobic tank for 48 h and counted. Repeat 3 times for each dilution. The viable bacteria counting formula was used to count, and the results were expressed in the form of colony value per gram of fecal wet weight to calculate the total number of colonies (CFU/g).

#### FDA microbial activity

FDA with the characteristic that can freely penetrate into and out of the complete cell membrane and be decomposed by lipase in the cell to produce polar fluorescein that can produce fluorescence was used to measure the microbial activity at 490 nm wavelength. FDA mixed acetone to prepare a 2 mg/mL FDA storage solution, which was stored in a refrigerator at 2–8 ℃ away from light. The FDA storage solution was transferred into PBS (pH 7.6) to prepare a concentration of 10 μg/mL of FDA reaction solution. Transfer 2 ml of FDA reaction solution and 50 μL sample diluent in a sterile test tube, and repeat 3 times. The sample was shaken at 24 ℃ for 90 min, and the reaction was terminated by adding 2 mL of acetone. The OD was measured with an ultraviolet spectrophotometer at λ490 nm. The definition of 1 microbial activity unit is the enzyme required that 1 g sample reacts for 90 min to produce 1 μmol of fluorescein.

#### Mucus barrier

The fresh colon at 1 cm from the rear end of the mouse cecum was dissected and sectioned with hematoxylin–eosin (HE) staining and alcian blue—periodic acid-Schiff (AB–PAS) staining. The histological morphology of the mouse colon was observed by an optical microscope, and the sections were digitally scanned. The images were processed by Caseviewer: C.V 2.3 system and the morphological changes of colonic mucosa were compared. 10 visual fields of different samples were randomly selected to count and calculate the number of goblet cells and the thickness of the mucus layer of the intestinal mucosa.

Muc2 in colonic tissue of mice was detected by enzyme-linked immunosorbent assay (ELISA). The intestinal tissue about 3 cm from the back of the cecum of mice was cut by surgery, weighed, labeled, and quick-frozen in dry ice. After grinding frozen tissue, tissue lysate was added to prepare intestinal tissue homogenate samples. The tissue samples were detected according to the steps of the mouse Muc2 ELISA kit. Mouse Muc2 ELISA kit is provided by Jiangsu Sofia Medical Technology Co., Ltd (20200397).

#### Amino acid transferases and blood lipids

The mice fasted for 12 h before sampling, and eyeball blood was collected into the procoagulant tube. The whole blood of mice was centrifuged at 3000 R/min^−1^ for 10 min, and the serum was taken for standby. Amino acid transferases in serum samples were determined by the automatic biochemical instrument, such as glutamate pyruvic transa (GPT), glutamic oxaloacetic transaminase (GOT), alkaline phosphatase (ALP), and γ-glutamyltransferase (GGT). Blood lipids in serum samples were determined by the automatic biochemical instrument, such as serum total cholesterol (TC), triacylglycerol (TG), high-density leptin cholesterol (HDL-C), and low-density leptin cholesterol (LDL-C).

#### Full-length PCR amplication

The bacterial DNA was extracted according to the steps of the HiPure Soil DNA Mini Kit (D3142-03). The concentration of DNA was determined by NanoDrop ND-2000 (Thermo Fisher Scientific, Waltham, MA, US), and the qualified DNA was amplified by PCR. The PCR amplication primers of the bacterial full-length 16S rRNA genes:

forward: 27F (5′-AGRGTTYGATYMTGGCTCAG-3′).

reverse: 1492R (5′-RGYTACCTTGTTACGACTT-3′).

Sample-specific16-bp barcodes were incorporated into the primers for multiplex sequencing. PCR reaction procedure: (a) 3 min at 95 ℃; (b) 25 cycles × (30 s at 95 ℃; 30 s at 56 ℃; 60 s at 72 ℃); (c) 5 min at 72 ℃. PCR amplicons were puried with Agencourt AMPure Beads (Beckman Coulter, Indianapolis, IN) and quantied BY the PicoGreen dsDNA AssayKit (Invitrogen, Carlsbad, CA, USA).

#### PacBio HiFi sequencing

The PCR products were end-repaired and spliced to generate a library by SMRTbell Template Prep Kit 1.0-SPv3 and accurately quantified by Qubit. Agilent 2100 was used to detect the size of the inserted fragment, and the expected DNA was sequenced on the PacBio platform. Sequencing was performed using the PacBio platform with DNA/Polymerase Binding Kit 3.0 (PacBio). Software SMRT link V8 0 was used to preprocess and filter the original PacBio sequencing output data to obtain circular consensus sequences (CCS). The clean CCS sequence was filtered by removing the CCS primer and unqualified length. Finally, the intestinal mucosa samples with an average length of CCS less than 1400 dpi were excluded for subsequent analysis to ensure the accuracy of the data.

#### Taxonomic and biomarker analysis

Qiime2 software (qiime. ORG) was used to cluster the clean CCS sequences of all samples with 97% identity. RDP classifier Bayesian algorithm was used to classify 97% of the OTU representative sequences at similar levels (with a confidence threshold of 0.8), and OTU annotation was compared with the Silva database. Mothur software (http://www.mothur.org/wiki/) calculated alpha diversity in this study, and the difference in alpha diversity between groups of mice was analyzed by the density curve in R v4.1.0. A significant difference in beta diversity between groups were tested using a one-way analysis of variance followed by post hoc Tukey honestly significant difference at a 95% confidence level. The composition of dominant microbiota was performed by GraphPad Prism v9.0.0, and the differences between the two groups were analyzed by the Wilcoxon rank-sum test. LDA effect size (LEfSe) analysis was used to find out the biomarkers in sample division (http://huttenhower.sph.harvard.edu/galaxy). Metastat analysis was used to detect significant microbial community differences between the two groups (http://metastats.cbcb.umd.edu/detection.html).

#### Functional analysis of microbiota metagenome

Functional profiling was performed using PICRUSt2 (Phylogenetic Investigation of Communities by Reconstruction of Unobserved States) v2.0.0-b with built-in EC/MetaCyc and KEGG/KO databases. The functional abundance of the sample is predicted based on the abundance of marker gene sequences in the sample. The platform inferred the metabolic pathways by using MinPath to obtain the abundance data of metabolic pathways. MetaCyc and KEGG pathway abundances were further analyzed using statistical analysis of Omic Share profiles (http://omicshare.com/tools/) and R v4.1.0 ggplot2 package. The correlation coefficient was calculated by using the cor () function in R v3.1.3. According to the *P* ≤ 0.05 and the correlation coefficient* R* ≥ 0.7, the relationship network between mucosal microbiota and amino acid metabolic function was constructed by using Cytoscape v3.9.0.

#### Metabonomic amino acid metabolites standard curve and limit of quantitation

Weigh an appropriate amount of 23 amino acid standards and prepare single standard mother liquor with methanol or water. The profiling of amino acid information and concentration points can be referred to (Additional file 1: Table S1). A liquid Chromatograph Mass Spectrometer (LC–MS) was used to detect each working standard solution and draw the standard curve. ACQUITY UPLC BEH C18 chromatographic column (2.1 × 100 mm, 1.7 μm) was set to 40 ℃ and a 5 μL standard solution was added. Mobile phase A-10% methanol–water (containing 0.1% formic acid), B-50% methanol–water (containing 0.1% formic acid). Gradient elution conditions were 0 ~ 6.5 min, 10 ~ 30% B; 6.5 ~ 7 min, 30 ~ 100% B; 7 ~ 8 min, 100% B; 8 ~ 8.5 min,100 ~ 10% B; 8.5 ~ 12.5 min, 10% B. Flow rate: 0 ~ 8.5 min, 0.3 mL/min; 8.5 ~ 12.5 min, 0.3 ~ 0.4 mL/min. The mass spectrometric condition was electrospray ionization (ESI) source, using positive ion ionization mode. The ion source temperature was 500 ℃, the ion source voltage was 5500 V, the collision gas was 6 psi, the air curtain gas was 30 psi, and the atomization gas and auxiliary gas were 50 psi. Multiple response monitoring (MRM) was used for scanning. The ions for quantitative analysis can be referred to (Additional file 2: Table S2). The concentration of the standard solution was taken as the abscissa and the area ratio of the working standard solution to the internal standard peak was taken as the ordinate to investigate the linear range and draw the standard curve (Additional file 3: Table S3). Eight standard solution samples reprocessed to determine intra-day and inter-day precision, repeatability, and recovery can be referred to (Additional file 3: Table S3).

#### Amino acid quantitative profiling

Transfer the mouse serum sample into a 2 ml EP tube and dilute it with 10% formic acid methanol solution ddH2O (1:1. V: V) solution. The diluted sample of 100 μL is added to a double isotope internal standard with a concentration of 100 ppb μL vortex oscillation for the 30 s. The supernatant was filtered with a 0.22 μmol/L membrane, and the filter solution was detected. All samples were analyzed quantitatively according to the established sample pretreatment and instrumental analysis methods. The data set was scaled by the “pheatmap” package in R v3.3.2 to export the hierarchical cluster diagram of relative quantitative values of amino acid metabolites. The metabolomics data were scaled by autoscaling, mean centering, and scaled to unit variance (UV), and then subjected to multivariate statistical analysis of partial least squares discriminant analysis (PLS-DA) and orthogonal partial least squares discriminant analysis (OPLS-DA) by using “ropls” package of R v3.3.2. The differential metabolites were screened according to the statistically significant *p*-value and fold change. Z-score was converted based on the content of metabolites and was used to measure the variety of metabolites at the same level.

#### Bioinformatics analysis

The co-occurrence network of intestinal mucosal microbiota was constructed to interpret the core bacteria in the relationship. According to *Spearman’s* correlation coefficient of intestinal mucosal microbiota, the positive or negative correlation between different microbial communities was investigated. *Pearson* correlation analysis was used to construct the relationship network of intestinal mucosal microbiota targeted amino acid metabolism in mice, to integrate the relationship between intestinal mucosal microbiota species and serum amino acid metabolites. The relationship pairs were screened in the conditions of the correlation coefficient R ≥ 0.7 and FDR < 0.05. According to the ranking of species abundance or metabolite concentration in all samples, Cytoscape v3.9.0 software was used to perform the correlation network of intestinal mucosal microbiota. The neural network algorithm was used to distribute the network nodes. The cor. test () function of R v3.1.3 was used to statistically test the correlation analysis of amino acid metabolites, and the heat map of amino acid metabolites was exported by using the “pheatmap” package of R v3.1.3 to reveal the synergy of metabolite variety. To explain the relationship between intestinal mucosal microbiota structure and amino acid metabolic environment, redundancy analysis (RDA) was used to study the multiple linear regression between microbiota response variables and explanatory variables of amino acid metabolites. The length of the arrow represents the constraint effect of different environmental variables exerted on sample distribution in two-dimension. Procrustes analysis was used to study the consistency of microbiota abundance and amino acid metabolome. RDA and Procrustes analysis were performed by using “vegan” and “ggplot2” packages of R v4.1.0. Random forest analysis was performed with the “randomForest” package of R v4.1.0. A mathematical model based on key bacteria and amino acid metabolome based on random forest variables was used to predict Muc2 content in the sampling group. Linear regression analysis was used to determine the quantitative relationship between colon Muc2 concentration and the A mathematical model.

#### Statistics

The continuous data conform to the normal distribution and was expressed by mean ± SEM. If the variance was homogeneous, the difference between the two groups was expressed by an independent sample t-test. One-way ANOVA was used for multi-group comparison, and the LSD test was used for multi-group pairwise comparison. If it does not conform to the normal distribution, the nonparametric rank-sum test was used. The rate of each group was expressed as a percentage (%), and the rate between groups was compared by the Chi-square test. Inspection level α = 0.05. *P* < 0.05 indicates significant difference; *P* < 0.01 indicates that the difference is highly significant.

## Results

### Intestinal microbiota disorder aggravates the injury of the intestinal mucus barrier induced by cold and humid environmental stress in mice

#### Effects of intestinal microbiota disorder on the morphology of intestinal mucus layer and Muc2 protein induced by cold and humid environment stimulation

**Fig. 2:**
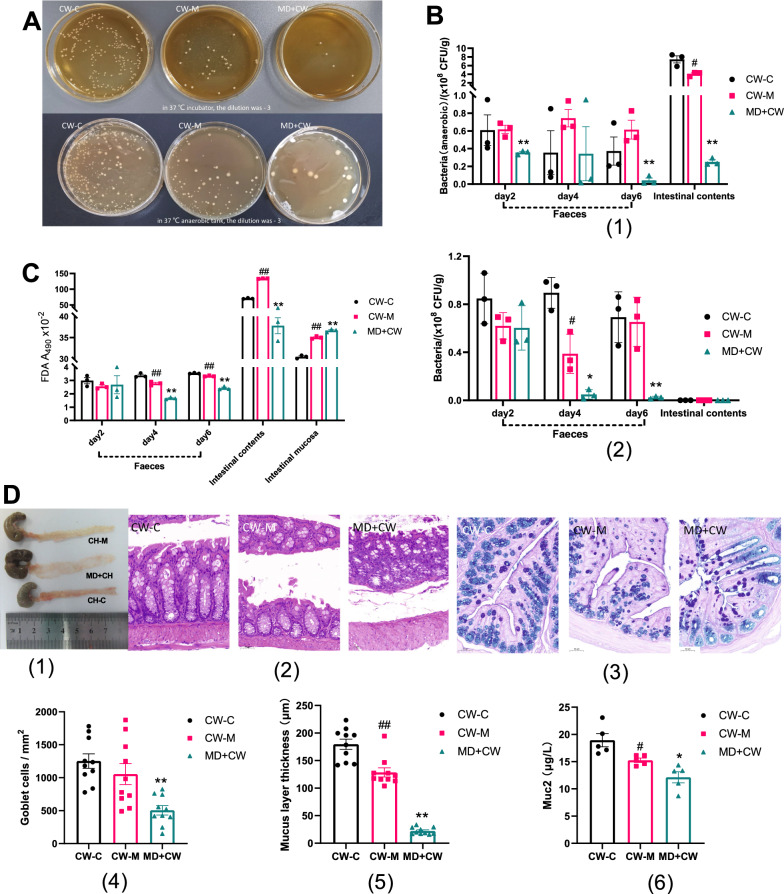
Intervention of intestinal microbiota disturbance on intestinal mucus barrier in cold and humid environmental stressed mice. **A** Intervention of intestinal microbiota disorder modeling on culturable bacteria in intestinal of mice with cold and humid environmental stress. On the 6th day of modeling, the bacterial culturing of fecal samples (10 times dilution) of CW-C group, CW-M group, and MD + CW group. up, in 37 ℃ incubator; down, in 37 ℃ anaerobic tank; the dilution was—3. **B** Effect of intestinal microbiota disorder modeling on the number of culturable bacterial colonies in feces, intestinal contents, and intestinal mucosa. (1) in 37 ℃ incubator; (2) in 37 ℃ anaerobic tank. **C** Effect of intestinal microbiota disorder modeling on FDA in feces, intestinal contents, and intestinal mucosa. **D** Effect of intestinal microbiota homeostasis on the intestinal mucus layer. (1) dissect longitudinally colonic tissue and expose the inner layer of mucosa; (2) Hematoxylin–eosin staining of colon tissue, 20 × 10; (3) AB-PAS staining of colon tissue, 20 × 10; the number of goblet cells (4), the thickness of mucus layer (5), and Muc2 (6) in intestinal tissue. CW-C, normal control group; CW-M, cold and humid environmental stress treatment group; MD + CW, microbiota disorder + cold and humid environmental stress treatment group. CW-C vs. CW-M, #*P* < 0.05, ##*P* < 0.01. CW-M vs. MD + CW, *P < 0.05, **P < 0.01

The intestinal microbiota disorder modeling on the 6th day could reduce the culturable bacteria in feces samples (Fig. 2A). The anaerobic (or facultative) bacteria colonizing in intestinal contents, cultured in an anaerobic environment, decreased significantly in the cold and humid environmental stress (*P* < 0.05), but it is not statistically significant in the fecal bacteria, and the culturable bacteria in MD + CW group decreased highly significantly in the intestinal microbiota disorder modeling for 2 and 6 days (*P* < 0.01) (Fig. 2^2^B1). In the standard culture environment, there were few culturable bacteria in intestinal contents, but in the MD + CW group, the culturable bacteria in feces decreased significantly on the 4th and 6th days with the intestinal microbiota disorder modeling (*P* < 0.05) (Fig. 2B2). From the 4th day to the end of cold and humid environmental stress the FDA in feces decreased highly significantly (*P* < 0.01), and the FDA in intestinal contents and mucosa increased highly significantly (*P* < 0.01); meanwhile, the intestinal microbiota disorder modeling highly significantly reduced the FDA of feces and contents, but highly significantly increased the FDA of the intestinal mucosa (*P* < 0.01) (Fig. 2C). The bacterial culture and FDA microbial enzyme activity showed that the intervention in this study could cause intestinal microbiota disorder in mice.

In the CW-M group, the intestinal mucosa was continuously broken, the subepithelial space was obvious, accompanied by the shedding of the top of local villi, and the rupture of the mucus layer; and in the MD + CW group, the submucosa and lamina propria of intestinal tissue were separated, the muscle layer was edematous, the arrangement structure of villi was disordered or missing, the neutral mucin and acidic mucin were seriously reduced, and the mucus layer was significantly thinned or disappeared (Fig. 2D1–3). Mucin is the main substance constituting the intestinal mucus barrier, Muc2 is the main protein constituting the mucus layer of intestinal tissue, and intestinal epithelial goblet cells are the main place for mucin secretion. Cold and humid environmental stress significantly reduced the thickness of the mucus layer and Muc2 in intestinal tissue (*P* < 0.05), and the disturbance of intestinal microbiota aggravated the significant reduction of goblet cells, mucus layer thickness, and Muc2 in intestinal tissue of mice with cold dampness stress (*P* < 0.01) (Fig. 2D4–6).

**Fig. 3:**
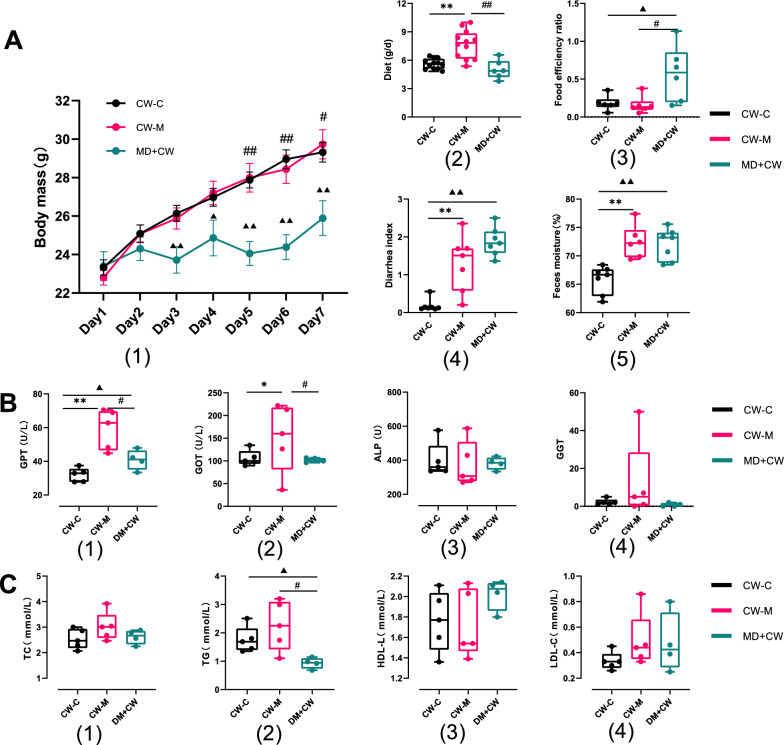
Intervention of intestinal microbiota disorder on GI disorders in cold and humid environmental stressed mice. **A** Effect of intestinal microbiota disorder on GI symptoms. **B** Effect of the intestinal microbiota disorder on serum amino acid transferase. **C** Effect of the intestinal microbiota disorder on bio-chemical serum lipid. CW-C, normal control group; CW-M, cold and humid environmental stress treatment group; MD + CW, microbiota disorder + cold and humid environmental stress treatment group. CW-C vs. CW-M, ^*^P < 0.05, ^**^P < 0.01. CW-M vs. MD + CW, ^#^*P* < 0.05, ^##^*P* < 0.01. CW-C vs. MD + CW, ^▲^*P* < 0.05, ^▲▲^*P* < 0.01

**Fig. 4:**
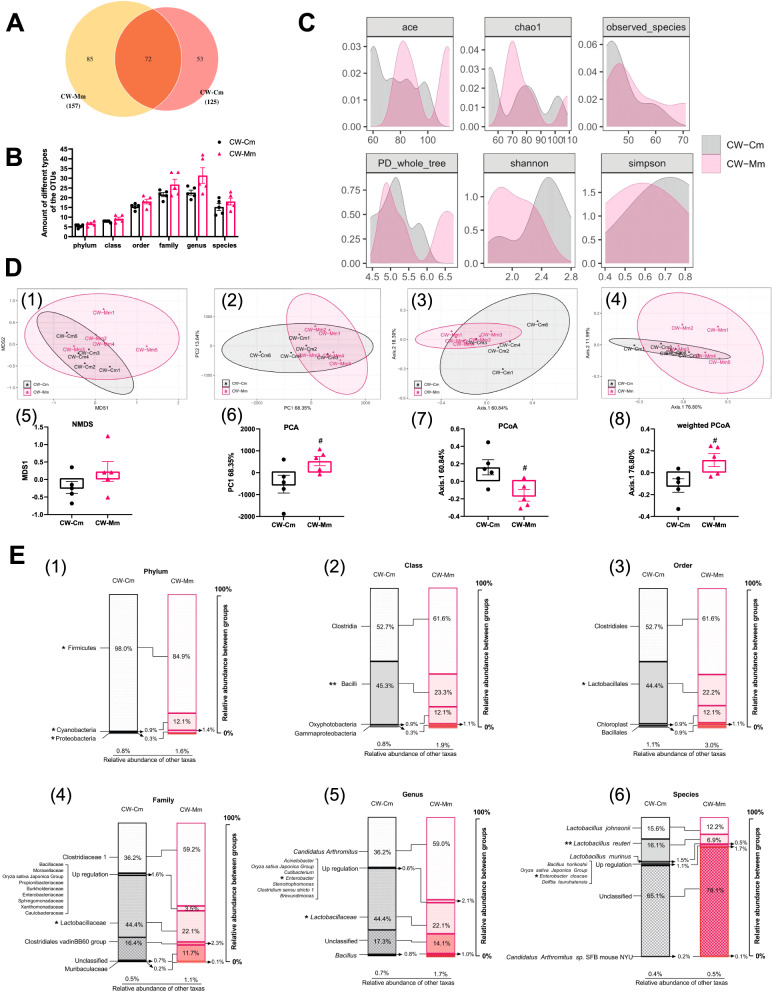
OTU annotation, gene richness, and microbial compositional changes. **A** OTU classification VEEN diagram. **B** The classification of OTU at the level of phylum, class, order, family, genus, and species. **C** Distribution illustration of samples gene representative of OTU number in community (ace index), community richness (chao1 index), species number in community (observed species index), phylogenetic tree distance in community (PD whole tree index), and community diversity (Shannon index and Simpson index). **D** Intestinal mucosal microbiota enterotype of cold and humid environmental stressed mice samples. Non-metric multidimensional scaling (NMDS) plot (1), principle component analysis (PCA) plot (2), principal coordinates analysis (PCoA) plot (3), and weighted PCoA plot (4) represent cold and humid environmental stressed mice samples highlighted by intestinal mucosal microbiota enterotype. **E** Difference analysis of dominant microbiota composition at the level of phylum (1), class (2), order (3), family (4), genus (5), and species (6). CW-Cm, intestinal mucosa samples of the normal control group; CW-Mm, intestinal mucosa samples of cold and humid environmental stress treatment group. CW-Cm vs. CW-Mm, Wilcoxon signed-rank test, *P < 0.05, **P < 0.01

#### The intestinal microbiota disorder aggravates the GI disorders of mice stimulated by cold and humid environmental stress

Cold and humid environmental stress significantly increased the diet, diarrhea index, and fecal water content (*P* < 0.05), but there was no significant difference in body mass (Fig. 3A). Intestinal microbiota disorder significantly reduced the body mass of environmental stress intervention mice and significantly increased the food efficiency ratio, diarrhea index, and fecal water content (*P* < 0.05) (Fig. 3A). It was found that cold and humid environmental stress significantly reduced the abdominal and anal temperature of mice by measuring with an ultraviolet thermometer (*P* < 0.05) (Additional file 5: Fig. S1). Cold and humid environmental stress significantly increased serum amino acid transferase such as GPT and GOT in mice (*P* < 0.05), and in the cold and humid stress environment, the intestinal microbiota disorder significantly decreased serum GPT, GOT, and blood lipid TG in mice (*P* < 0.05) (Fig. 3B, C).

### Intestinal mucus barrier injury induced by cold and humid environmental stress disturbed the microecology of intestinal mucosal microbiota in mice

#### The changes in diversity and the composition of intestinal mucosal microbiota trans-formed by intestinal mucus barrier injury induced by cold and humid environmental stress

After preprocessing and filtering the original sequencing output data, the primers of CCS were removed to obtain the NonPrimers sequence, and the filtered sequence samples with a length of 1300—1600 BP were reserved for subsequent analysis. The sample information can be referred to (Additional file 4: Table S4). The bacterial DNA sequences of intestinal mucosal microbiota are divided by 210 OTUs in 97% similarity in this study (Fig. 4A); however, there was no significant difference in OTUs between the CW-C and CW-M groups (Fig. 4B). The dilution curve was constructed with the number of sequences and OTUs in the random sampling of sequences, and the accumulation curve indicated that OTUs reached asymptote with the increase of sampling (Additional file 6: Fig. S2). The density curve demonstrated that the peaks of microbiota Shannon and Simpson indexes of alpha diversity were separated (Fig. 4C). The confidence intervals of NMDS, PCA, PCoA, and weighted PCoA analysis of intestinal mucosal microbiota in the CW-CM group and CW-MM group were significantly separated, and the differences in PCA, PCoA, and weighted PCoA were statistically significant (*P* < 0.05) (Fig. 4D). The results demonstrated that cold and humid environmental stress could reshape the composition structure of intestinal mucosal microbiota in mice.

The intervention effect of cold and wet environment stress on intestinal mucosa microbiota in mice was analyzed by the composition structure of dominant bacteria (relative abundance > 0.1%). Classification from phylum to family level, cold and humid environmental stress significantly reduced the relative abundance of Firmicutes – Bacilli – Lactobacillales – Lactobacillaceae (*P* < 0.05), and significantly increased the relative abundance of Cyanobacteria and Proteobacteria (*P* < 0.05) in the intestinal mucosa of mice (Fig. 4E1–4). In terms of genus classification, the relative abundance of *Lactobacillus* decreased significantly (*P* < 0.05), and the relative abundance of *Enterobacter* increased significantly (*P* < 0.05) (Fig. 4E5). The relative abundance of *Lactobacillus reuteri* decreased highly significantly (*P* < 0.01), and the relative abundance of *Enterobacter cloacae* increased significantly (*P* < 0.05) in species classification (Fig. 4E6).

#### Potential biomarkers of intestinal mucosa in mice in response to cold and humid environmental stress

**Fig. 5:**
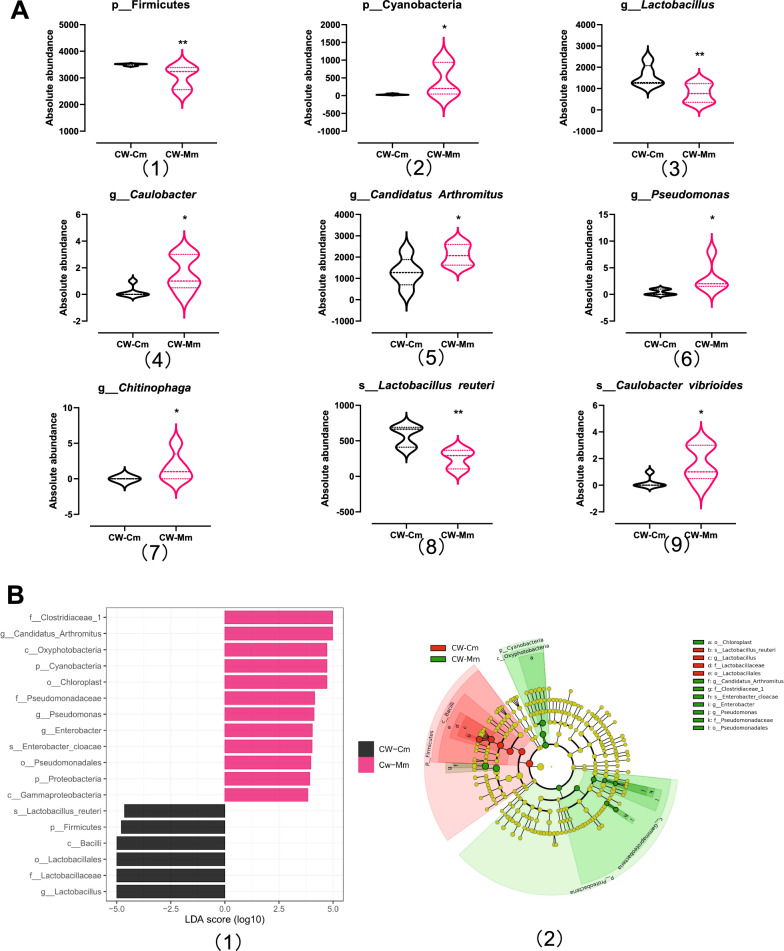
Intestinal mucosa microbiota biomarkers of cold and humid environmental stress. **A** Mothur-metastats analysis of differential microbiota between CW-Cm and CW-Mm. *P < 0.05, **P < 0.01. **B** Linear discriminant analysis (LDA) Effect size (LEfSe) plot revealed the taxonomic biomarkers associated with cold and humid environmental stress. The threshold for LDA score was 2 (*P* < 0.05). (1) Histogram of LDA scores to identify differentially abundant microbiota. (2) Cladogram generated from the LEfSe analysis indicating the phylogenetic distribution from phylum to species of the microbiota. CW-Cm, intestinal mucosa samples of normal control group; CW-Mm, intestinal mucosa samples of cold and humid environmental stress treatment group

In this study, Metastat was used to analyze the microbiota with significant differences, and LEfSe was used to screen the bacteria or species that can significantly distinguish the intestinal mucosal microbiota of normal mice and cold and humid environmental stressed mice, that is, the potential biomarkers in response to cold and humid environmental stress. Metastat analysis showed that at the phylum level, the abundance of Firmicutes decreased highly significantly (*P* < 0.01), and the abundance of Cyanobacteria increased significantly (*P* < 0.05); at the genus level, the abundance of *Lactobacillus* decreased highly significantly (*P* < 0.01), while the abundance of *Caulobacter*, *Candidatus Arthromitus*, *Pseudomonas* and *sphingosphaga* increased significantly (*P* < 0.05); at the species level, the abundance of *Lactobacillus reuteri* decreased highly significantly (*P* < 0.01), and the abundance of *Caulobacter vibrioides* increased significantly (*P* < 0.05) (Fig. 5A). There are 18 microbiotas with an LDA score greater than 2 in LEfSe analysis, which can be used as mouse mucosal microbial markers in response to cold and humid environmental stress (Fig. 5B1). The microbial markers are Firmicutes—Bacilli—lactobacillales—Lactobacillaceae—*Lactobacillaceae*—*Lactobacillus reuteri*, Cyanobacteria—Oxyphotobacteria—Chloroplast, Clostridiaceae 1—*Candidatus Arthromitus*, Proteobacteria—Gammaproteobacteria – *Escherichia* – *Enterobacter cloacae*, and Proteobacteria—Gammaproteobacteria—Pseudomonadales—Pseudomonadaceae – *Pseudomonas* (Fig. 5B2). The results indicated that the absolute dominant microbiota *Lactobacillus reuteri* had the greatest difference at all classification levels, so it can be used as a potential biomarker of the intestinal mucosal microbiota in response to cold and humid environmental stress.

#### The amino acid metabolic function of response microbiota to cold and humid environmental stress

**Fig. 6:**
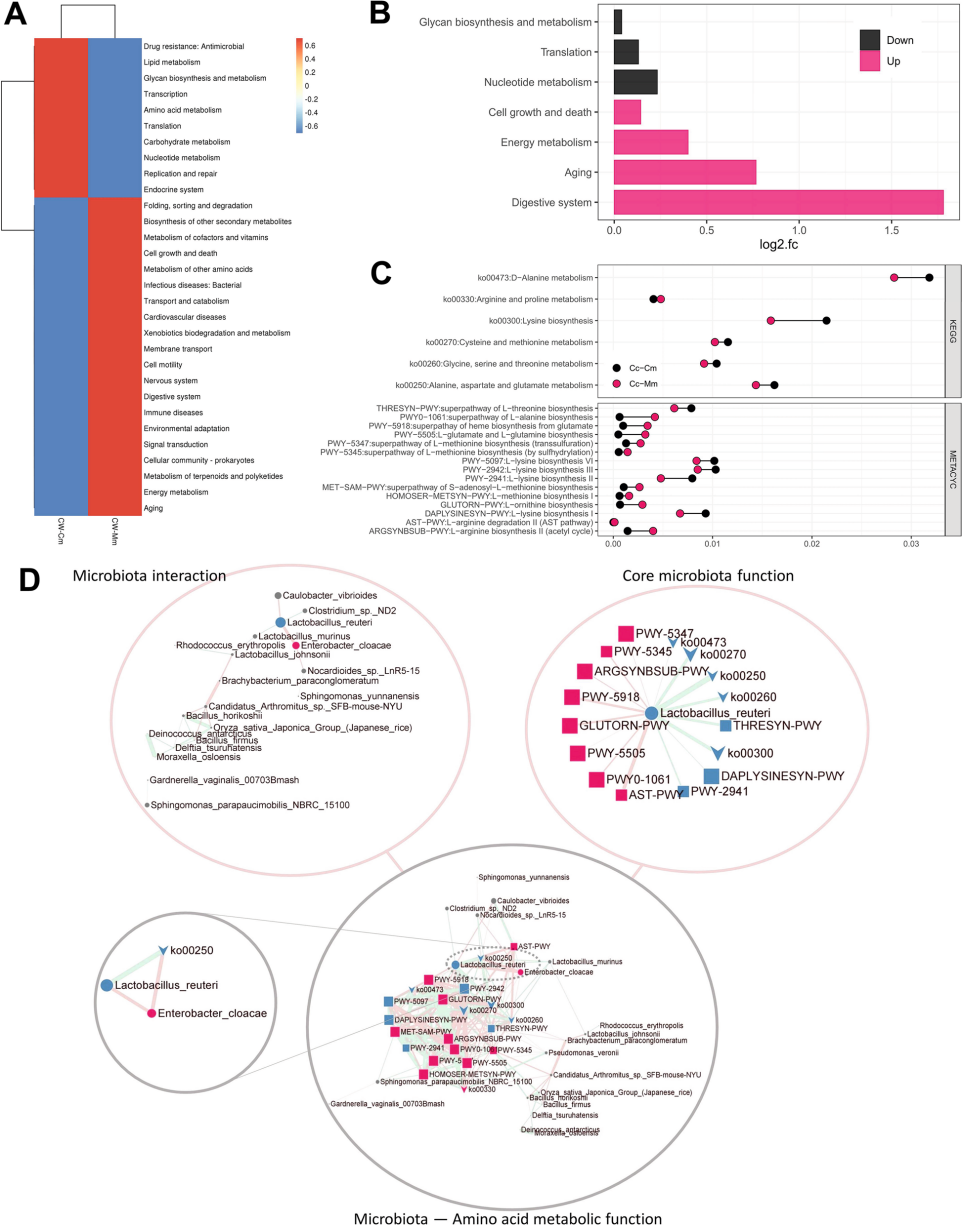
Amino acid metabolic functional annotation and intestinal community function network modeling of the altered microbiota.** A** Cluster analysis of KEGG secondary function. **B** KEGG secondary function difference analysis. **C** Amino acid metabolism pathway analysis of KO/KEGG and EC enzymes/MetaCyc. **D** The network modeling of microbiota and amino acid metabolic function. The microbiota interaction network based on microbiota species abundance and *Spearmen’s* correlation in all samples (R ≥ 0.7, FDR < 0.05). The core microbiota function network based on microbiota species abundance significance between cold and humid environmental stressed mice and normal control mice (Wilcoxon signed-rank test, FDR < 0.05) and *Spearmen’s* correlation in all samples (R ≥ 0.7, FDR < 0.05). The microbiota amino acid metabolic function network based on microbiota species abundance, KEGG pathway, MetaCyc pathway, and *Spearmen’s* correlation in all samples (R ≥ 0.7, FDR < 0.05). Circled, arrowed, and squared shapes are related to microbiota species, KEGG pathway, and MetaCyc pathway, respectively. Red and blue nodes are related to up-regulated and down-regulated abundance significance between cold and humid environmental stressed mice and normal control mice (Wilcoxon signed-rank test, FDR < 0.05), respectively; and gray circled nodes represent microbiota species with no difference. Based on R value of Spearmen correlation, the red and blue edges represent negative and positive correlation, respectively; and the width of the edge represents correlation coefficient; the greater the width, the stronger the correlation

Picrust2 docked the KEGG database and the MetaCyc database respectively and predicted 6307 KEGG homologous genes (including 755 up-regulated KOs and 163 down-regulated KOs, *P* < 0.05) and 1,947 EC enzyme labels of microbiota (including 307 up-regulated ECs and 69 down-regulated ECs, *P* < 0.05) (Additional file 6: Fig. S2). The cluster analysis of KEGG secondary function found that the cold and humid environmental stress reduced the abundance of the intestinal mucosal microbiota in mice with amino acid metabolism, notably the microbiota related to digestive system function significantly reduced with the largest fold change (FC > 1.5), indicating that the cold and humid environmental stress mainly affected the digestive system function of mucosal microbiota (Fig. 6A, B). The amino acid metabolism in the KEGG database indicated that ko00300, ko00473, ko00250, ko00270, ko00260, and ko00330 were the main amino acid metabolic pathways of cold and humid environmental stress intervention on intestinal mucosal microbiota (Fig. 6C). The MetaCyc database showed that the microbiota genes enriched in DAPLYSINESYN-PWY, PWY-2941, PWY-2942, PWY-5097, and THRESYN-PWY pathways decreased significantly (*P* < 0.05), and the microbiota genes enriched in ARGSYNBSUB-PWY, PWY0-1061, PWY-5918, PWY-5347, and PWY-5505 increased pathways significantly (*P* < 0.05) (Fig. 6C).

Intestinal-community modeling reveals the role of bacterial amino acid metabolism on cold and humid environmental stress. The dominant bacteria *Lactobacillus reuteri* and *Enterobacter cloacae* had a negative correlation and responded to the cold and humid environmental stress (Fig. 6D). The alanine aspartate and glutamate metabolic pathway (ko00250) was the main amino acid metabolic pathway of *Lactobacillus reuteri* more relevantly, the ko00250 was associated with *Lactobacillus reuteri* and *Enterobacter cloacae* in the core relationship network (Fig. 6D). It is concluded that in this study the increase of host amino acid transferase induced by the cold and humid environmental stress was related to the metabolism of alanine aspartate glutamate in microbiota, notably *Lactobacillus reuteri*.

### Profiling the amino acid metabolism changes in mice treated with cold and humid environmental stress

#### The overall difference in amino acid metabolome response to cold and humid environmental stress in mice

**Fig. 7:**
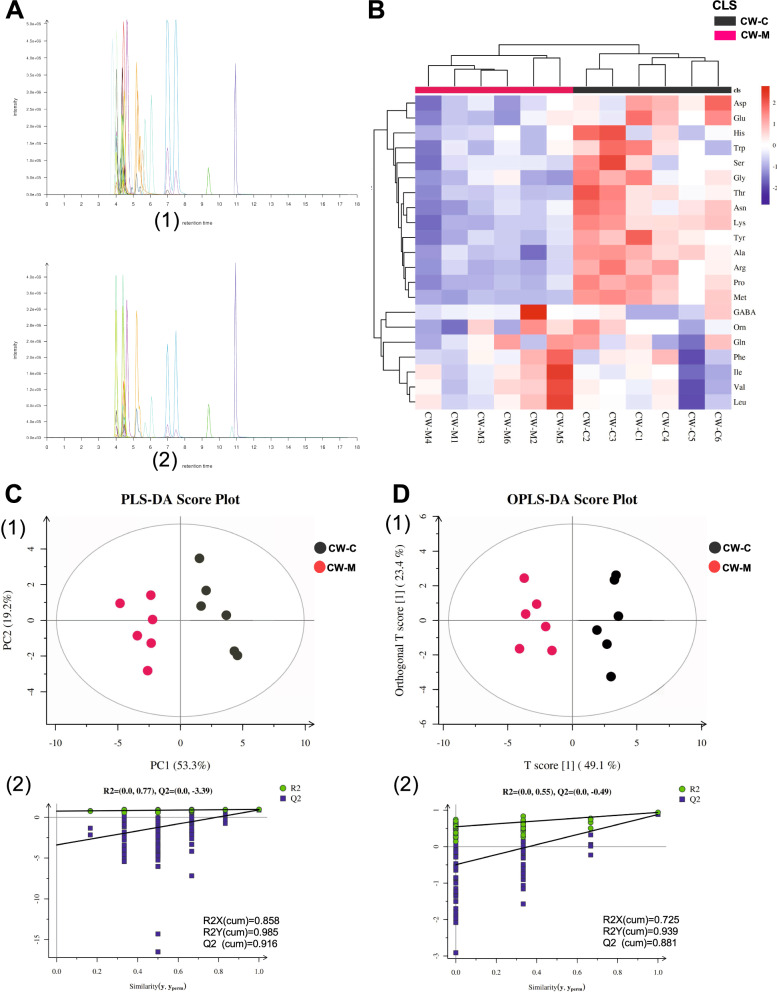
Overall difference of amino acid metabolome. **A** LC–MS total ion chromatogram (TIC). (1) Mixed standard TIC; (2) Samples TIC. **B** Cluster analysis of amino acid metabolism. **C** PLS-DA analysis; **D** OPLS-DA analysis. CW-C, normal control group; CW-M, cold and humid environmental stress treatment group

LC–MS results showed that the chromatographic peaks of mixed standard total ion chromatography (TIC) and sample TIC overlap, indicating that the corresponding intensity and retention time of each color spectrum peak was consistent, and the variation error margin was narrow in the whole experimental process (Fig. 7A). Hierarchical clustering was used to expound on the metabolic pattern of serum amino acids in cold and humid environmental stressed mice, which showed the metabolic patterns of L-aspartic acid (Asp) and L-glutamic acid (Glu) were clustered into one group in the top two, indicating the consistency of the two metabolic patterns (Fig. 7B). The dimension reduction model was established by partial least squares discriminant analysis (PLS-DA) (Fig. 7C1) and orthogonal partial least squares discriminant analysis (OPLS-DA) (Fig. 7D1) in the supervised pattern recognition, and the result was indicative of a clear separated distribution of amino acid metabolism in the cold and humid environmental stress mice and normal control mice on the x-axis. The explanatory degrees of the dependent variable (RX2), independent variable (RY2), and predictability (Q2) of the model cross-validated were greater than 0.5 and less than 1, indicating that the model has good repeatability and small intragroup difference, and a large overall difference in amino acid metabolism between groups (Fig. 7C2 and Fig. 7D2).

#### Quantification of amino acid metabolites and screening of differential metabolites in cold and humid environmental stressed mice

**Fig. 8:**
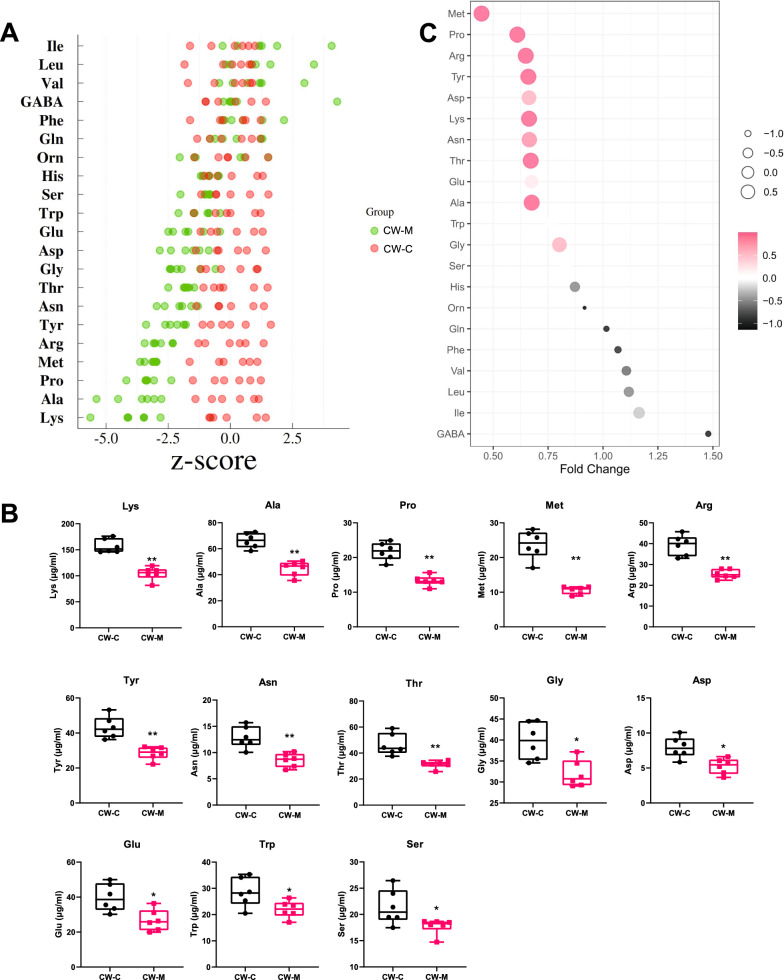
Quantitative analysis of amino acid metabolites and screening of differential amino acid metabolites. **A** Conversion of standard score (Z-score) of amino acid metabolite content in mice serum. **B** The differential amino acid metabolites between the two groups based on an independent sample t-test. *P < 0.05, **P < 0.01. **C** The differential amino acid metabolites between the two groups based on fold change. The point size and colour represent the *P* value. CW-C, normal control group; CW-M, cold and humid environmental stress treatment group

The standard score (Z-score) conversion was performed for the amino acid metabolite quantification in serum samples to measure the metabolite content in the same dimension (Fig. 8A). Combined with Z-score and quantitative analysis, it was found that cold and humid environmental stress highly significantly reduced Ala, Pro, Thr, Lys, Met, Arg, Tyr, and Asn (*P* < 0.01), and significantly reduced Gly, Asp, Glu, Ser, and Trp (*P* < 0.05) in mice (Fig. 8B). The amino acid metabolite with the largest fold change (FC) was γ 4-aminobutyric acid (GABA); and even more remarkably, Ala, Glu, and Thr were statistically significant with the large FC (Fig. 8C). The results indicated that Ala, Glu, Thr, and GABA are the main amino acid metabolic profiles that participate in the response mechanism of cold and humid environmental stress.

### Contribution of host amino acid metabolic environment and intestinal mucosal microbiota to intestinal mucus barrier injury induced by cold and humid environmental stress in mice.

#### Modeling of relationship network between intestinal mucosal microbiota and targeted amino acid metabolism

**Fig. 9:**
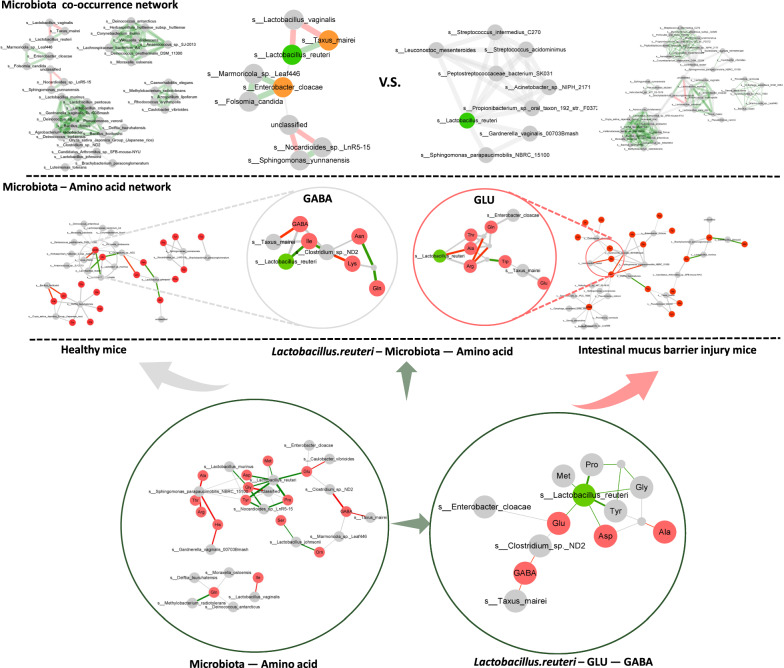
Integrative correlation network between intestinal mucosal microbiota and targeted amino acid metabolism. Low part: Network of intestinal mucosal microbiota and serum amino acid metabolism in mice based on the *Pearson* correlation between all samples. Mid part: Network of intestinal mucosal microbiota and serum amino acid metabolism in mice, based on the *Pearson* correlation of groups in the normal control group (i.e., Healthy mice) and the cold and humid environmental stress treatment group (i.e., Intestinal mucus barrier injury mice), respectively. Up part: Microbiota Co-occurrence network, based on microbiota species abundance and the *Pearson* correlation of groups in the normal control group (i.e., Healthy mice) and the cold and humid environmental stress treatment group (i.e., Intestinal mucus barrier injury mice), respectively. The integrative correlation network is based on microbiota species abundance, metabonomic amino acid quantification and Pearson correlation in all samples (R ≥ 0.7, FDR < 0.06). Red, grey, and green circled shapes are related to the amino acid, microbiota species, and *Lactobacillus reuteri*, respectively; and yellow circled shapes in microbiota Co-occurrence network represent the significance differential microbiota between cold and humid environmental stressed mice and normal control mice (Wilcoxon signed-rank test, FDR < 0.05). The green and red edges represent positive and negative correlations, respectively (R ≥ 0.7, FDR < 0.05); and the grey edges represent a hint of significance (R ≥ 0.7, 0.05 ≤ FDR < 0.06). The width of the edge represents the correlation coefficient; the greater the width, the stronger the correlation

We integrated the HiFi sequencing genomics and serum amino acid metabolomics data using metabolic modeling and construct an integrative correlation network giving insight into key microbial species linked with intestinal mucus barrier injury and amino acid metabolism response to the cold and humid environmental stress. *Spearman’s* correlation was used to determine the associations between the abundance of intestinal microbiota and amino acid metabolites (Fig. 9). Microbiota—amino acid network revealed that the abundance of *Lactobacillus reuteri* is significantly decreased and positively correlated with amino acid metabolites Glu and GABA in all mice. Personalized microbial species metabolic modeling of microbiota and amino acid network revealed *Lactobacillus reuteri* contribution to amino acid metabolites GABA observed in healthy mice, and amino acid metabolites Glu observed in mice with intestinal mucus barrier injury induced by cold and humid environmental stress. *Lactobacillus reuteri* colonized in the intestinal mucosa of healthy mice contributed greatly to the microbiota co-occurrence network, which was positively correlated with *Taxus mairei*, negatively correlated with *Lactobacillus vaginalis*, and had a certain correlation with *Enterobacter cloacae*. The regulation of *Lactobacillus reuteri* on intestinal mucosal microbiota co-occurrence network in model mice decreased, meanwhile the regulatory effect of *Lactobacillus reuteri* on intestinal mucosal microbiota co-occurrence network was lost in mice with intestinal mucus barrier injury induced by cold and humid environmental stress.

#### Prediction of intestinal mucus Muc2 in mice by host amino acid metabolic environment and intestinal mucosal microbiota

**Fig. 10:**
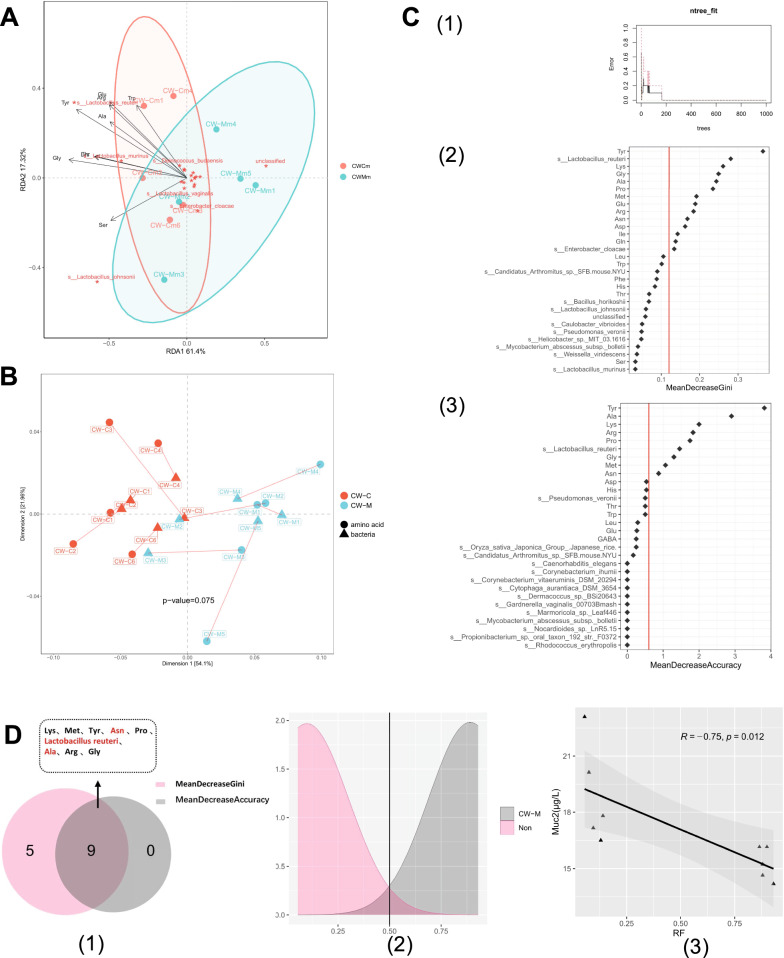
Muc2 prediction model based on machine learning random forest method. Correlation between intestinal mucosal microbiota and targeted amino acid metabolism: **A** RDA analysis and **B** Procrustes Analysis. **C** A random forest classifier on the intestinal mucosal microbiota and serum amino acid quantification training sets to realize the cold and humid environmental stressed mice classification. (1) Decision tree learning algorithm. The markers were selected as key biomarkers by random forest method based on mean decrease Gini (2) and mean decrease accuracy (3). The red line illustrates the number of key markers in the discovery set. **D** Establishment of the prediction model. (1)A mathematical model based on 9 key markers was used to predict the sampling group. (2) The probability plot indicates the prediction probability. The red color indicates the predicted group and the grey color indicates the non-predicted group. (3) Taxa from the random forest model to establish the mathematical model could utilize the relative abundance of intestinal mucosal microbiota and serum metabonomic amino acid quantification to predict intestinal tissue Muc2 content with R^2^ of 0.75 and *P* of 0.012. CW-C, normal control group; CW-M, cold and humid environmental stress treatment group. CW-Cm, intestinal mucosa samples of normal control group; CW-Mm, intestinal mucosa samples of cold and humid environmental stress treatment group

Although cold and humid environmental stress perturbs microbiota and amino acids in mice, whether the difference in intestinal mucosal microbiota is associated with these amino acids changes, remains unknown. Thus, we performed RDA and Procrustes analysis to evaluate the relationship between the host amino acid metabolic environment and intestinal mucosal microbiota. RDA analysis shows that the information content of the x-axis (61.4%) and y-axis (17.32%) exceeds 75%, indicating that it reflects the overall state of the sample. The microbiota composition of cold and humid environmental stressed mice were affected by the host amino acid metabolic environmental factors and was remarkably separated from healthy mice in the second quadrant. The microbiota that contributes greatly to this separation is *Lactobacillus reuteri*, followed by unclassified bacteria and *Lactobacillus murinus*. The arrow of environmental factors indicates that the environmental factors of amino acid metabolism that have a great impact on the microbiota are Tyr, Glu, and Arg (Fig. 10A). Procrustes analysis showed the correlation between intestinal mucosal flora abundance and targeted amino acid metabolomics a fell marginally short of significance (*P* = 0.07) (Fig. 10B).

We have shown that cold and humid environmental stress regulates the host amino acid metabolic balance and intestinal mucosal microbiota. However, it is difficult to distinguish their importance to the intestinal mucus barrier injury. Here, the key bacteria and amino acid metabolites associated with cold and humid environmental stress were identified by the random forest method, and the Muc2 in intestinal tissue prediction model was established. By applying fivefold cross-validation on a random forest model, among individual samples at the species classification, in the discovery phase, 500 decision trees were generated resulting in optimal markers that were selected based on the lowest mean error rate and standard deviation, and the significance level of the model is less than 0.001, and the accuracy of classification is 100% (Fig. 10C). Thus, we selected the 9 shared top candidates as the key marker in response to cold and humid environmental stress. These include Lys, Met, Tyr, Asn, Pro, *Lactobacillus reuteri*, Ala, Arg, and Gly (Fig, 10D1). Furthermore, given the key position of these 9 key markers significantly correlated with cold and humid environmental stress, within the microbiota community and host amino acid metabolic environment, we built a prediction model that can recognize the target samples (Fig, 10D2). The model could predict intestinal mucus Muc2 with an adjusted R^2^ of 75.0% (Fig, 10D3). The results support the effects of host amino acid metabolic environment and intestinal mucosal microbiota on intestinal mucus barrier injury, notably the colonization of *Lactobacillus reuteri* in the intestinal mucosa.

## Discussion

Stress, including psychological, environmental, and physical stressors, has varied biological effects which are increasingly recognized as including modulation of com-mensal microorganisms residing in the gut [23]. The cold and humid environmental stress is a comprehensive interference factor for physiology and psychology. Growing evidence has demonstrated that cold and humid stress triggers GI disorders [3, 4]. It is generally accepted that the environmental stress reaction is an adaptive response necessary for the survival of the organism adapts. There is clear evidence proving that the dynamic adaptive adjustment of gut microbiota and metabolites endows the host with the plasticity of thermogenesis regulation in response to temperature fluctuations, which is very important for thermostatic animals to improve their survival suitability in low temperature environment [24]. A study found that cold stress altered the compositions and functions of the cecal microbiota, which is related to host thermogenesis and insulin resistance [25]. Once the adaptive responses are destroyed, the disease will occur, causing GI disorders. Notably, despite the fundamental importance of gut microbiota in adapting to environmental stress and causing GI disorders, relatively little attention has been paid to the influence and mechanism of cold and humid environmental stress on the intestinal microbiota. Here, the disorder of gut microbiota leads to more damage to the intestinal mucus barrier in mice with cold and humid environmental stress, which may be related to the reduction of colonization of *Lactobacillus reuteri* in intestinal mucosa and the imbalance of amino acid metabolisms such as Glu, Met, and Asp.

Recent researchers have found that factors that affect the intestinal mucus barrier, such as food intake [26, 27], inflammatory bowel diseases (IBDs) [28, 29], and Helicobacter pylori infection [30], are key determinants of microbiome composition, therefore directly influence host GI health, and has been reviewed extensively elsewhere. However, environmental factors known to influence the intestinal mucus barrier have been comparatively neglected in the microbiome field. In this study, we found intestinal micro-biota disorder aggravated intestinal mucosal damage and GI disorders caused by cold and humid environmental stress, and confirmed the role of intestinal microbiota in cold and humid environmental stress. Environmental factors such as temperature and humidity are of fundamental importance in controlling microbial growth and activity [31, 32], and the changes in the host’s microbiota diversity and composition are caused by environmental factors that will affect the intestinal mucus barrier. Secretory mucin Muc2, a glycosylated protein, is the most secreted mucin secreted by the intestinal tract. For the complex bidirectional interaction between host glycans and gut microbes, the intestinal mucus barrier injury allows commensal and pathogenic microorganisms to reach the intestinal epithelium, thereby leading to infection and inflammation [6]. Inflammation activates peroxisome proliferator-activated receptor gamma (PPAR γ) to induce the energy metabolism of colon cells and oxygen availability of intestinal microbiome, and promote goblet cells to secrete mucus to expel pathogenic bacteria or toxins [33]. The mucin degradation by gut bacteria sulfatases that utilize distal colonic mucin O-glycans is an important process for both normal microbial gut colonization and diseases such as inflammatory bowel disease [8].

This study found that there was no significant difference in alpha diversity of intestinal mucosal microbiota in the cold and humid environmentally stressed mice, but there was a significant difference in microbial community beta diversity, that is, cold and humid environmental stress can interfere with the composition of intestinal mucosal microbiota in mice. The composition of the gut microbiota undergoes changes from the mucosal to the luminal/faecal side, while the composition of microbiota colonized in the intestinal mucosa is more stable [34] and more sensitive to reflect the microbiota ectopic colonization in the gut [35], which is most closely related to host immunity [36]. The intestinal mucosa depends on the innate immune function to sense the microorganisms in the cavity and send signals accordingly to produce a protective immune response, hence the intestinal mucosal immune system achieves a delicate balance between the tolerance of symbiotic organisms and obvious inflammation to resist pathogens [37]. Using the PacBio HiFi sequencing, we characterized key symbiont dominant bacteria *Lactobacillus reuteri* of intestinal mucosa involved in cold and humid environmental stress. A study has demonstrated that *Lactobacillus reuteri*, a probiotic effective in maintaining intestinal homeostasis, stimulates intestinal epithelial proliferation with increased Muc2 expression in animal intestinal tissue [38]. The mucin degradation is the key prebiotic function of *Lactobacillus reuteri*. A study has found that glyceraldehyde-3-phosphate dehydrogenases (cw-GAPDH) in *Lactobacillus reuteri* cell walls increase the ability to adhere to the host’s intestinal mucus layer to enhance the probiotic effects [39].

The gut microbiota influences the host cold stress response and associated sequelae, thereby implicating the gut microbiota as an important mediator of host health [23]. Cold exposure is an important risk factor for hypertension, and the gut microbiota can regulate blood pressure by transplanting faecal bacteria [31]. In addition, angiotensin-converting enzyme 2 regulates blood pressure and can regulate intestinal amino acid homeostasis and the gut microbiome [40]. It is consistent with our research results. In this study the serum amino acid transferases such as GPT and GOT in cold and humid environmentally stressed mice increased significantly, while after high-dose mixed antibiotics interfered with gut microbiota, GPT and GOT decreased significantly in response to cold and humid environmental stress, indicating that the gut microbiota can adapt to cold and humid environmental stress by regulating the host's amino acid metabolism. Further integrating the analysis of intestinal mucosal microbiota amino acid metabolic function and serum targeted amino acid metabolome in cold and humid environmental stressed mice, it was found that there is a plausible relationship between the metabolic functions of Asp, Ala, and Glu of intestinal mucosal microbiota and the differential amino acid metabolites GABA and Glu in the host. The inspiring results are concerned with the effects of amino acid neurotransmitters metabolism, especially glutamate metabolism, mediated by intestinal mucosal microbiota homeostasis on the intestinal mucus barrier and GI disorders caused by cold and humid environmental stress. Amino acid neurotransmitters are generally considered the critical modulators regulating the gut-brain axis (GBA) which is a bilateral communication network between the GI tract and the central nervous system and plays a significant role in GI physiology [41, 42]. Glutamate including the metabolites such as GABA and Gln involved in the circuitry of the enteric nervous system widely regulates the diverse GI behavior patterns for maintaining GI homeostasis in physiologic and pathologic conditions [43, 44]. Moreover, there is evidence that circulating glutamine and glutamine/glutamate are shaped by the gut microbiome [45]. A recent study also showed that transplantation of the “cold microbiota” increased thermogenesis, which might have been due to the interaction of the intestinal microbiota with host neurotransmitters[25, 46].

Although a study has shown that GABA (and its precursor glutamine) increases mucin expression [47], the role of intestinal microbiota remains unknown. Our study found that the cold and humid environmental stress reduced the glutamate including the metabolites such as GABA and Gln in mice, which may be one of the main reasons for intestinal mucus barrier injury and GI dysfunction. More studies show that Ala-Gln supplementation can protect the intestinal mucosal barrier and alleviate intestinal inflammation [48–50]. The synergistic effect of glutamate metabolism between host and intestinal microbiota in response to cold and humid environmental stress is related to *Lactobacillus reuteri* in our study. Glutamate and GABA formations were strain-dependent, and *Lactobacillus reuteri* can accumulate glutamate and GABA [51, 52].

Our findings provide evidence supporting the link between gut microbiota and intestinal mucus barrier caused by cold and humid environmental stress. Most importantly, we explained the microbiota mechanism of cold and humid environmental stress from a new perspective, which enhances our understanding of intestinal mucus barrier and amino acid metabolism. However, due to the limited current availability of intestinal mucosa microbiota studies, further studies on the crosstalk between amino acid neurotransmitters and intestinal mucin response to cold and humid environmental stress are warranted. In this study, the metabolic function of bacteria is based on the predictive algorithm, and the analysis of co-metabolism of host and bacteria lacks validation experiments in vitro, so the internal relationship between bacterial metabolism and mucus barrier and mucin-modified biological enzymes is not explained. Additional investigation into adaptive changes of intestinal microbiota and extensive physiological effects on cold and humid environmental stress is required. We believe the present study could provide a new perspective for interpreting the relationship between GI disorders caused by comprehensive stress interference and microbial dysbiosis.

### Supplementary Information

**Additional file 1: **Table S1. LC-MS metabonomic amino acid information and concentration points.

**Additional file 2: Table S2.** The ions for LC-MS metabonomic amino acid quantitative analysis.

**Additional file 3: Table S3.** The standard curve and quality control for LC-MS metabonomic amino acid quantitative analysis.

**Additional file 4: Table S4.** The sample CCS information for intestinal mucosa PacBio HiFi sequencing microbial genomics. CW-Cm, intestinal mucosa samples of normal control group; CW-Mm, intestinal mucosa samples of cold and humid environmental stress treatment group.

**Additional file 5: Figure S1.** Intervention of cold and humid environment stress on body temperature of mice. **A** Anal temperature. **B** Core temperature. * *P*＜0.05, ** *P*＜0.01, *** *P*＜0.001. CW-C, normal control group; CW-M, cold and humid environmental stress treatment group.

**Additional file 6: Figure S2.** Dilution curve constructed with the number of sequences and OTUs. CW-Cm, intestinal mucosa samples of normal control group; CW-Mm, intestinal mucosa samples of cold and humid environmental stress treatment group.** Figure S3.** Notes of KEGG database and MetaCyc database. **A** KOs. **B** EC enzyme labels. Red and black dots are related to up-regulated and down-regulated KOs/ECs, respectively.

## Data Availability

All data relevant to the study are included in the article or uploaded as supplementary information. Sequencing data have been uploaded to NCBI SRA with accession number PRJNA846958.
