# Supplementary material for: Intestinal mucosal microbiota mediate amino acid metabolism involved in the gastrointestinal adaptability to cold and humid environmental stress in mice
Source: Microb Cell Fact. 2024 Jan 24;23:33. doi: 10.1186/s12934-024-02307-2 (PMC10809741; doi:10.1186/s12934-024-02307-2)
Supplement: Supplementary file 1 — Additional file 1: Table S1. LC-MS metabonomic amino acid information and concentration points. [file 12934_2024_2307_MOESM1_ESM.docx]

**Additional file 1: Table S1. LC-MS metabonomic amino acid information and concentration points.**

| Id | Abbreviation | Amino acid | CSA |
| --- | --- | --- | --- |
| 1 | Gly | Glycine | 56-40-6 |
| 2 | Ala | L-Alanine | 56-41-7 |
| 3 | GABA | 4-Aminobutyric acid | 20791 |
| 4 | Ser | L-Serine | 56-45-1 |
| 5 | Pro | L-Proline | 147-85-3 |
| 6 | Val | L-Valine | 72-18-4 |
| 7 | Thr | L-Threonine | 72-19-5 |
| 8 | Ile | L-Isoleucine | 73-32-5 |
| 9 | Leu | L-Leucine | 61-90-5 |
| 10 | Asn | L-Asparagine | 70-47-3 |
| 11 | Orn | L-Ornithine hydrochloride | 3184-13-2 |
| 12 | Asp | L-Aspartic acid | 56-84-8 |
| 13 | Hcy | DL-Homocysteine | 454-29-5 |
| 14 | Gln | L-Glutamine | 56-85-9 |
| 15 | Lys | L-Lysine | 56-87-1 |
| 16 | Glu | L-Glutamic acid | 56-86-0 |
| 17 | Met | L-Methionine | 63-68-3 |
| 18 | His | L-Histidine | 71-00-1 |
| 19 | Phe | L-Phenylalanine | 63-91-2 |
| 20 | Arg | L-Arginine | 74-79-3 |
| 21 | Tyr | L-Tyrosine | 60-18-4 |
| 22 | Trp | L-Tryptophan | 73-22-3 |
