# Supplementary material for: Intestinal mucosal microbiota mediate amino acid metabolism involved in the gastrointestinal adaptability to cold and humid environmental stress in mice
Source: Microb Cell Fact. 2024 Jan 24;23:33. doi: 10.1186/s12934-024-02307-2 (PMC10809741; doi:10.1186/s12934-024-02307-2)
Supplement: Supplementary file 2 — Additional file 2: Table S2. The ions for LC-MS metabonomic amino acid quantitative analysis. [file 12934_2024_2307_MOESM2_ESM.docx]

**Additional file 2: Table S2.The ions for LC-MS metabonomic amino acid quantitative analysis.**

| **ID** | **amino acid** | **Parent ion** | **Daughter ion** | **DP** | **EP** | **CE** | **CXP** |
| --- | --- | --- | --- | --- | --- | --- | --- |
| 1 | Gly | 76.026 | 30.000 | 41 | 10 | 17 | 8 |
| 2 | Ala | 90.097 | 44.100 | 31 | 10 | 17 | 2 |
| 3 | GABA | 104.051 | 87.000 | 41 | 10 | 15 | 6 |
| 4 | Ser | 106.054 | 60.100 | 26 | 10 | 17 | 12 |
| 5 | Pro | 116.092 | 70.000 | 51 | 10 | 21 | 6 |
| 6 | Val | 118.148 | 72.000 | 41 | 10 | 13 | 14 |
| 7 | Thr | 120.098 | 102.000 | 36 | 10 | 15 | 6 |
| 8 | Ile | 132.052 | 85.900 | 20 | 10 | 30 | 15 |
| 9 | Leu | 132.077 | 85.900 | 56 | 10 | 17 | 16 |
| 10 | Asn | 133.055 | 116.000 | 61 | 10 | 23 | 8 |
| 11 | Orn | 133.084 | 70.100 | 41 | 10 | 23 | 6 |
| 12 | Asp | 134.052 | 88.000 | 31 | 10 | 15 | 8 |
| 13 | Hcy | 136.011 | 90.000 | 51 | 10 | 17 | 8 |
| 14 | Gln | 147.089 | 84.100 | 31 | 10 | 25 | 8 |
| 15 | Lys | 147.150 | 84.000 | 36 | 10 | 23 | 6 |
| 16 | Glu | 148.086 | 84.200 | 51 | 10 | 23 | 6 |
| 17 | Met | 150.101 | 132.900 | 41 | 10 | 13 | 12 |
| 18 | His | 156.076 | 110.000 | 56 | 10 | 19 | 10 |
| 19 | Phe | 166.102 | 120.100 | 56 | 10 | 19 | 10 |
| 20 | Arg | 175.103 | 70.000 | 66 | 10 | 31 | 6 |
| 21 | Tyr | 184.078 | 166.200 | 56 | 10 | 13 | 16 |
| 22 | Trp | 206.054 | 178.200 | 51 | 10 | 15 | 4 |
