# Supplementary material for: Intestinal mucosal microbiota mediate amino acid metabolism involved in the gastrointestinal adaptability to cold and humid environmental stress in mice
Source: Microb Cell Fact. 2024 Jan 24;23:33. doi: 10.1186/s12934-024-02307-2 (PMC10809741; doi:10.1186/s12934-024-02307-2)
Supplement: Supplementary file 3 — Additional file 3: Table S3. The standard curve and quality control for LC-MS metabonomic amino acid quantitative analysis. [file 12934_2024_2307_MOESM3_ESM.docx]

**Additional file 3: Table S3.** **The standard curve and quality control for LC-MS metabonomic amino acid quantitative analysis.**

| Id | Amino acid | Correlation coefficient | linear equation | QC Stability（%） | RSD Repeatability（%） |
| --- | --- | --- | --- | --- | --- |
| 1 | Gly | 0.9928 | y=0.0002235x + 0.007185 | 2.6 | ND |
| 2 | Ala | 0.9911 | y=0.001521x + 0.009119 | 1.57 | 9.36 |
| 3 | GABA | 0.9918 | y=0.001598x + 0.001859 | 9.47 | 9.92 |
| 4 | Ser | 0.9924 | y=0.002751x + 0.00808 | 0.64 | 12.85 |
| 5 | Pro | 0.99 | y=0.01172x + 0.008253 | 1.39 | 8.57 |
| 6 | Val | 0.994 | y=0.0064x + 0.009776 | 0.82 | 9.89 |
| 7 | Thr | 0.9926 | y=0.0005862x + 0.001542 | 1.69 | 13.89 |
| 8 | Ile | 0.9984 | y=0.001124x + 0.002134 | 0.94 | 14.71 |
| 9 | Leu | 0.9936 | y=0.007993x + 0.01252 | 1.52 | 11.46 |
| 10 | Asn | 0.9954 | y=4.034e-05x + 0.001084 | 8.67 | ND |
| 11 | Orn | 0.9918 | y=0.003031x + 0.004766 | 3.04 | ND |
| 12 | Asp | 0.991 | y=0.001429x + 0.001492 | 1.91 | 6.13 |
| 13 | Hcy | 0.9936 | y=0.0001951x - 0.001239 | NA | ND |
| 14 | Gln | 0.9957 | y=0.0007761x + 0.0009714 | 2.63 | 10.4 |
| 15 | Lys | 0.992 | y=0.001661x + 0.002166 | 3.96 | 13.63 |
| 16 | Glu | 0.9924 | y=0.002419x + 0.004507 | 1.65 | 7.19 |
| 17 | Met | 0.99 | y=0.001421x + 0.001376 | 1.08 | ND |
| 18 | His | 0.9908 | y=0.005236x + 0.007764 | 3.67 | ND |
| 19 | Phe | 0.9958 | y=0.0116x + 0.01654 | 1.15 | 9.46 |
| 20 | Arg | 0.9935 | y=0.005075x + 0.005389 | 4.12 | 13.97 |
| 21 | Tyr | 0.9966 | y=0.001664x + 0.004119 | 2.17 | ND |
| 22 | Trp | 0.9903 | y=0.0078x + 0.003635 | 2.1 | 8.91 |
