# Supplementary material for: Intestinal mucosal microbiota mediate amino acid metabolism involved in the gastrointestinal adaptability to cold and humid environmental stress in mice
Source: Microb Cell Fact. 2024 Jan 24;23:33. doi: 10.1186/s12934-024-02307-2 (PMC10809741; doi:10.1186/s12934-024-02307-2)
Supplement: Supplementary file 4 — Additional file 4: Table S4. The sample CCS information for intestinal mucosa PacBio HiFi sequencing microbial genomics. CW-Cm, intestinal mucosa samples of normal control group; CW-Mm, intestinal mucosa samples of cold and humid environmental stress treatment group. [file 12934_2024_2307_MOESM4_ESM.docx]

**Additional file 4: Table S4. The sample CCS information for intestinal mucosa PacBio HiFi sequencing microbial genomics.**

| **Sample** |  | **CCS** | **NonPrimers** | **Filtered** | **Average sequencing length** |
| --- | --- | --- | --- | --- | --- |
| CW-Cm1 |  | 12611 | 5944 | 3622 | 1478 |
| CW-Cm2 |  | 12154 | 10382 | 9968 | 1469 |
| CW-Cm3 |  | 14106 | 8500 | 6452 | 1461 |
| CW-Cm4 |  | 12115 | 7677 | 5750 | 1461 |
| CW-Cm5 |  | 14766 | 5434 | 3098 | 892 |
| CW-Cm6 |  | 13065 | 6995 | 6083 | 1460 |
| CW-Mm1 |  | 11044 | 7482 | 6489 | 1439 |
| CW-Mm2 |  | 9999 | 8507 | 8102 | 1453 |
| CW-Mm3 |  | 10242 | 7201 | 6088 | 1462 |
| CW-Mm4 |  | 13100 | 6634 | 4936 | 1453 |
| CW-Mm5 |  | 10853 | 7173 | 5795 | 1449 |
| CW-Mm6 |  | 11301 | 5967 | 2547 | 862 |
