# Supplementary figures and images for: Intestinal mucosal microbiota mediate amino acid metabolism involved in the gastrointestinal adaptability to cold and humid environmental stress in mice
Source: Microb Cell Fact. 2024 Jan 24;23:33. doi: 10.1186/s12934-024-02307-2 (PMC10809741; doi:10.1186/s12934-024-02307-2)

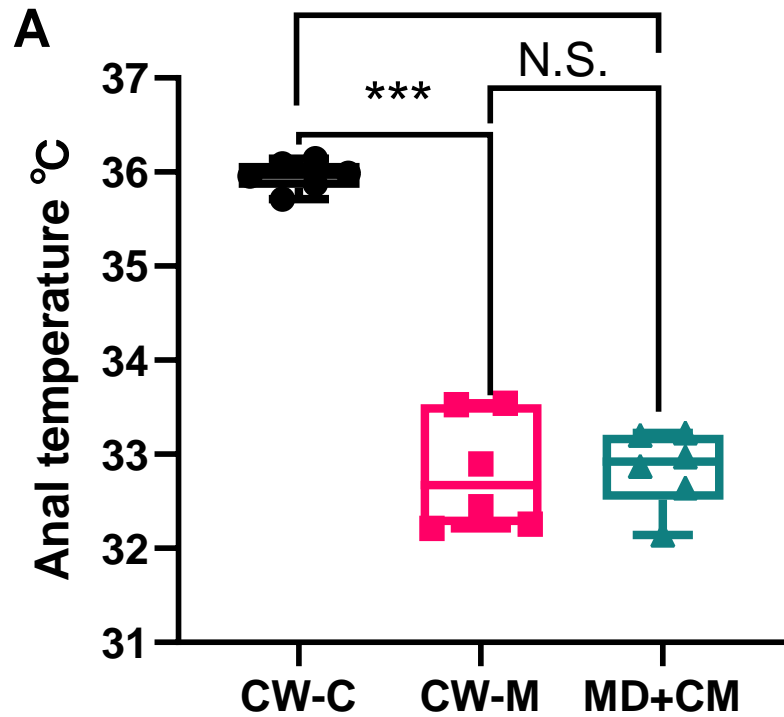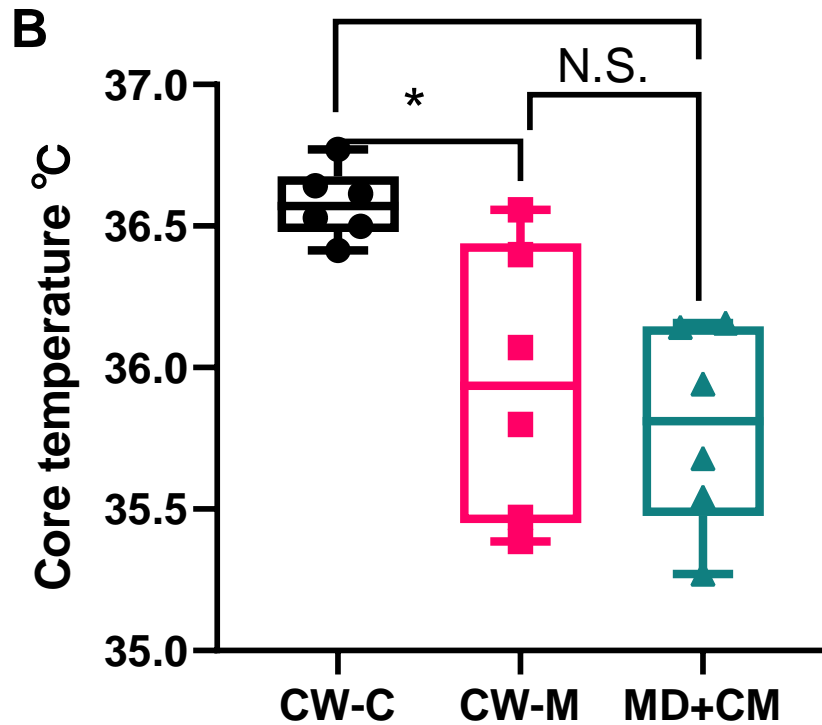

Supplement: Supplementary file 5 — Additional file 5: Figure S1. Intervention of cold and humid environment stress on body temperature of mice. A Anal temperature. B Core temperature. * P＜0.05, ** P＜0.01, *** P＜0.001. CW-C, normal control group; CW-M, cold and humid environmental stress treatment group. [file 12934_2024_2307_MOESM5_ESM.pdf]

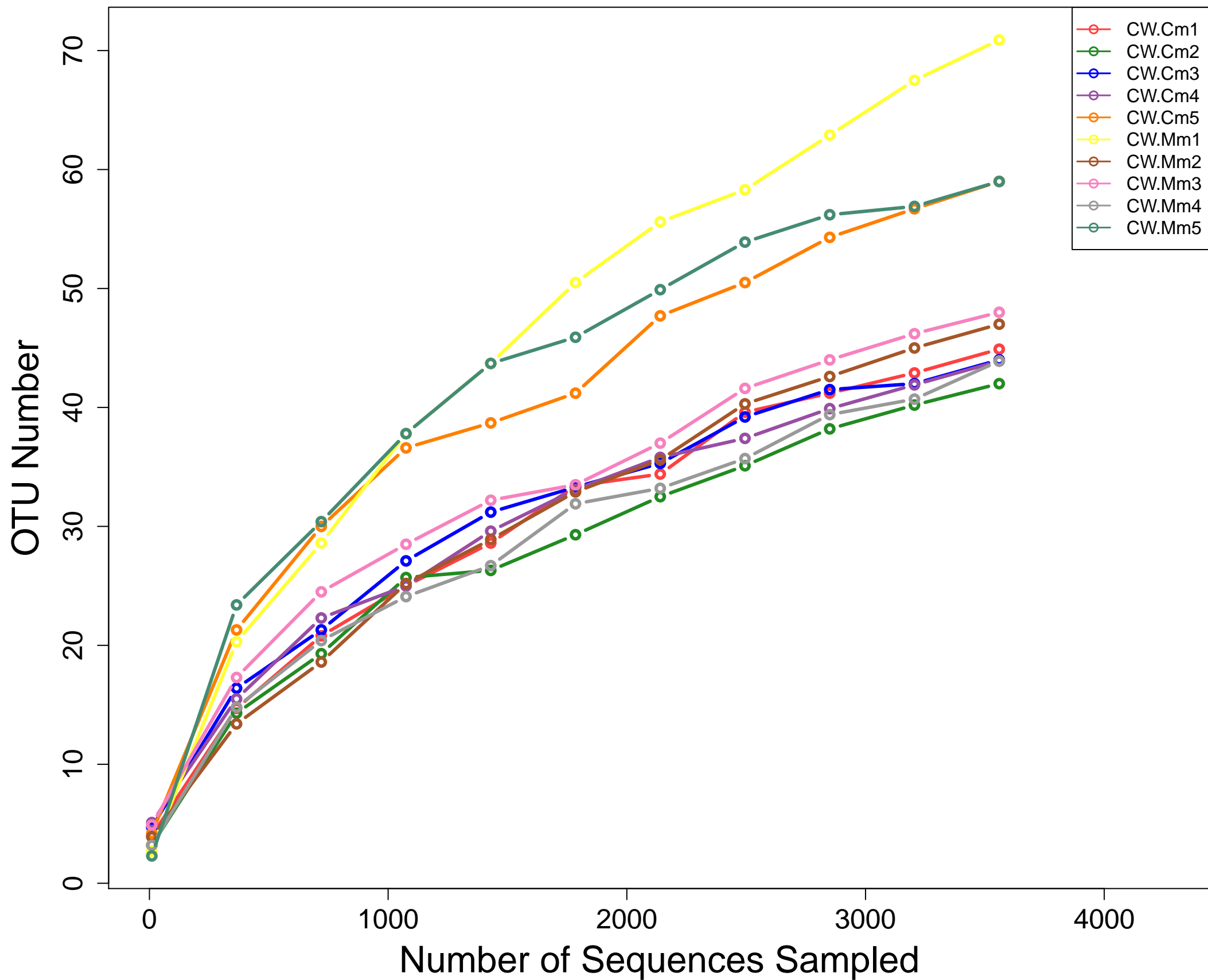

**A**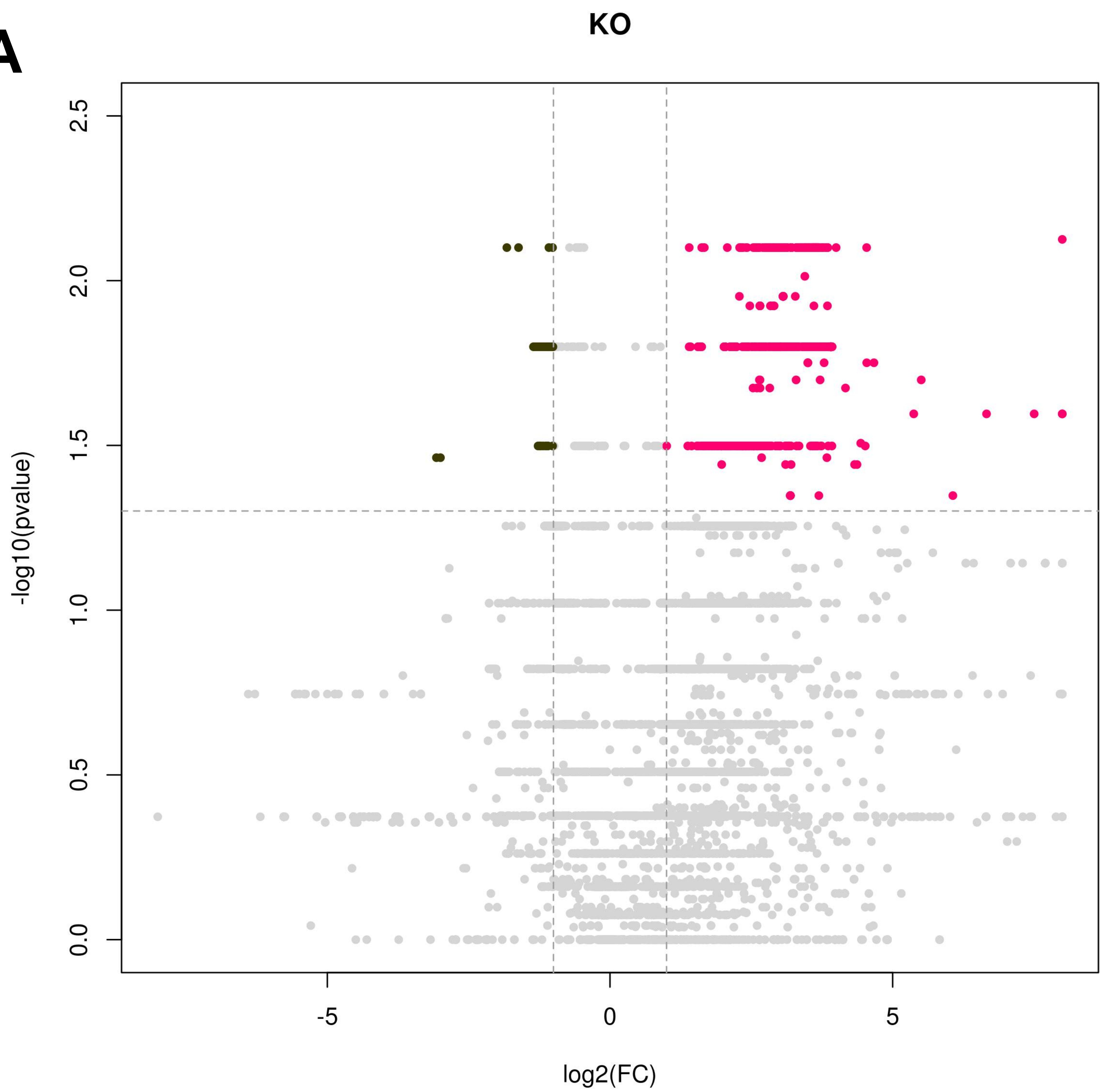**B**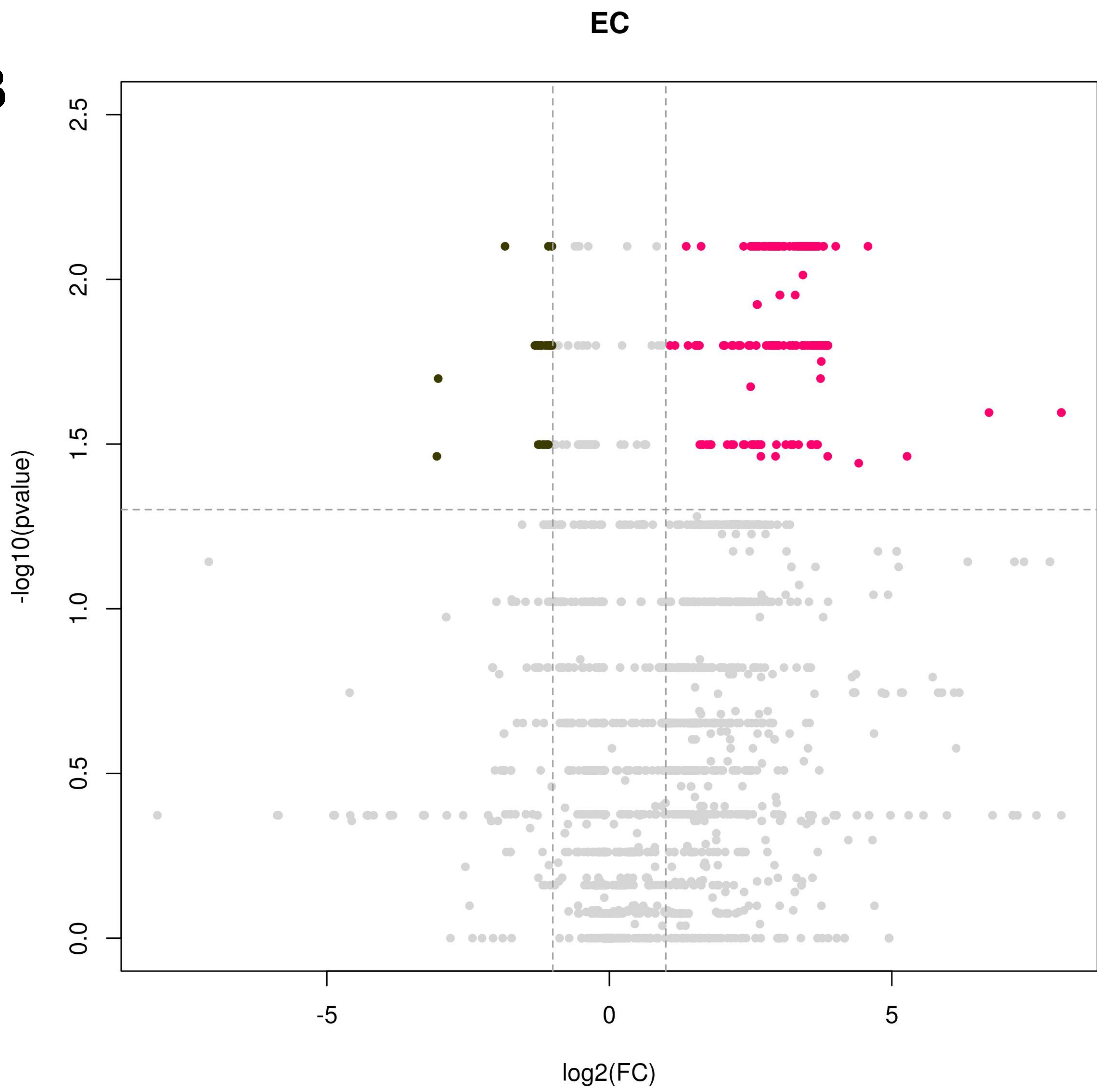

Supplement: Supplementary file 6 — Additional file 6: Figure S2. Dilution curve constructed with the number of sequences and OTUs. CW-Cm, intestinal mucosa samples of normal control group; CW-Mm, intestinal mucosa samples of cold and humid environmental stress treatment group. Figure S3. Notes of KEGG database and MetaCyc database. A KOs. B EC enzyme labels. Red and black dots are related to up-regulated and down-regulated KOs/ECs, respectively. [file 12934_2024_2307_MOESM6_ESM.pdf]
